# Investigating the Anti-Inflammatory Activity of *Juglans regia* Fresh Fruit Extract

**DOI:** 10.3390/foods15020368

**Published:** 2026-01-20

**Authors:** Lorenza Marinaccio, Eleonora Procino, Giulia Gentile, Stefano Pieretti, Angelo Cichelli, Adriano Mollica, Azzurra Stefanucci

**Affiliations:** 1 Department of Innovative Technologies in Medicine and Dentistry, University “G. d’Annunzio” of Chieti-Pescara, Via dei Vestini 31, 66100 Chieti, Italy; lorenza.marinaccio@unich.it (L.M.); angelo.cichelli@unich.it (A.C.); 2 Department of Pharmacy, University “G. d’Annunzio” of Chieti-Pescara, Via dei Vestini 31, 66100 Chieti, Italy; eleonora.procino@phd.unich.it (E.P.); giulia.gentile@phd.unich.it (G.G.); a.mollica@unich.it (A.M.); 3 National Centre for Drug Research and Evaluation, Istituto Superiore di Sanità, 00161 Rome, Italy; stefano.pieretti@iss.it; 4 SCM Nutraceutici Universitari srl, Strada degli Oliveti 73, 66100 Chieti, Italy

**Keywords:** inflammation, docking, polyphenols, abscisic acid

## Abstract

Numerous research works have tried to evaluate the correlation between inflammation and the onset of prostate cancer. Given the in vitro antioxidant power and the anti-proliferative effects on human prostate cancer cells shown by a *Juglans regia* L. fresh fruit extract, the aim of this work was the evaluation of its potential in the acute and chronic inflammatory states in vivo, revealing a strong anti-inflammatory activity. In the zymosan-induced edema formation assay, a light and non-significant edema reduction was shown. On the contrary, in the zymosan-induced thermal hyperalgesia assay, the reversion of hyperalgesia after the extract administration was determined. Moreover, in the formalin test, the extract caused a significant decrease in the licking time caused by the aldehyde, especially in the late phase. In silico, quercetin showed the best fit into the enzymatic pocket of AChE (docking score: −11.306 Kcal/mol). Neochlorogenic acid and ellagic acid gave the best docking scores on BChE (−10.292 Kcal/mol and −10.054 Kcal/mol, respectively). Abscisic acid showed a high binding affinity for the glucocorticoid receptor. Finally, quercetin and abscisic acid were quantified to complete the data by HPLC-DAD, giving 0.246 ± 0.003 mg/g of dried extract and 0.036 ± 0.004 mg/g of dried extract, respectively.

## 1. Introduction

*Juglans regia* L., belonging to Juglandaceae, is the scientific name of walnut, the most extensively outspread tree nut in the world. Walnuts are essential in human nutrition since the kernels are rich in proteins and oil [1,2]. Despite the absence of a description of walnut oil by the Committee on Fats and Oils of the Codex Alimentarius, it is produced in small quantities in some countries (France, Argentina, Spain, and Chile) and used mainly as salad dressing [3]. In traditional medicine, *J. regia* has been used for dermal inflammation, chronic eczema, or scrofula; in particular, the leaves have been applied topically in case of scalp itching, sunburn, dandruff, or superficial burns, and the kernels to treat inflammatory bowel disease in Iran [4].

The anti-inflammation and antinociceptive effects of different parts of *J. regia* were also evaluated. The anti-inflammatory and antinociceptive activities of aqueous and ethanolic extracts of *J. regia* leaves were determined by Hosseinzadeh et al., showing an antinociceptive effect via non-opioid receptors and an anti-inflammatory effect in acute and chronic inflammation models [5]. Mobashar et al. evaluated the effect of walnut leaf extracts on diverse arthritic models of chronic inflammation and acute inflammation. The ethanolic and *n*-hexane extracts showed anti-inflammatory and immunomodulatory properties, which improved the induced arthritis and edema. The reduction of joint inflammation could be due to the downregulation of TNF-α, IL-1β, IL-6, NF-κB, and COX-2 (pro-inflammatory markers) and the upregulation of IL-4 [6]. Fizeșan et al. investigated the antitussive, antioxidant, and anti-inflammatory activities of a *J. regia* septum extract, showing an antitussive effect that could be due to a cell protective effect and an anti-inflammatory effect. These outcomes could be related to the stimulation of the antioxidant enzyme systems and the decrease in IL-6 and CXC-R1 concentrations in lung tissue homogenates from the treated animals [7].

In our previous work, the polyphenolic profile of a *J. regia* fresh fruit extract was evaluated, and its in vitro antioxidant and enzyme inhibition activities, its antiproliferative effect on prostate cancer cells, and its in vivo effect on testosterone-induced benign prostatic hyperplasia (BPH) animal model were evaluated [8]. The studies mentioned above on the anti-inflammatory and antinociceptive effects of *J. regia* leaves and septum prompted us to investigate the potential anti-inflammatory and analgesic effects of this *J. regia* fresh fruit extract.

Moreover, there is scientific evidence regarding the potential role of prostate asymptomatic inflammation caused by microorganisms and the development of prostate cancer. Among these, Escherichia coli is the most frequently isolated microorganism found in patients suffering from bacterial prostatitis; furthermore, it was identified in BPH and prostate cancer tissues using culture-dependent and independent molecular techniques [9,10]. Actually, both environmental and hereditary factors are involved in the pathogenesis of prostate cancer: among the environmental factors, chronic inflammation gained attention due to several potential causes, e.g., infections, hormonal changes, and dietary aspects [10,11]. The evaluation of the correlation between prostatitis and the risk of developing prostate cancer was the topic of several studies, leading to both negative and positive results [12,13].

Considering all these findings, the determination of the anti-inflammatory effect of *J. regia* fresh fruit extract, having a dose- and time-dependent anti-proliferative effect on cancerogenic prostate cells, is even more interesting. Thus, the aim of this work is the determination of the in vivo anti-inflammatory and analgesic effects of *J. regia* fresh fruit extract and a possible molecular mechanism through in silico methods.

## 2. Materials and Methods

### 2.1. Plant Material and Reagents

The immature fruit of *J. regia*, also called green walnuts, was provided by a local vendor (Chieti, Italy). It was collected in May 2022 in Roccaspinalveti (Chieti, Italy) and stored for 1 week in a dark glass airtight container until its use. Ethanol puriss. (96.0–97.2%), water HPLC Plus, acetonitrile for HPLC (gradient grade, ≥99.9%), formic acid puriss. p.a. ⁓98% (T), and (+)-Abscisic acid analytical standard were all purchased from Sigma Aldrich (Milan, Italy). Quercetin dihydrate (analytical standard) was purchased from ChemCruz^®^ (Santa Cruz Biotechnology, Heidelberg, Germany).

### 2.2. Extraction Procedure

A maceration was performed (7 days, occasionally shaking) in a closed vessel, adding a mixture of ethanol/water 80:20 to the triturated plant matrix. The preparation of the sample and the extraction procedure were performed as previously described [8].

### 2.3. In Vivo Experiments

#### 2.3.1. Animals

All the experiments were conducted using male CD-1 mice (3–4 weeks, 25 g) (Harlan, Correzzana, Italy). They were located in colony cages held at standard conditions of temperature, light, and relative humidity for at least one week before the start of the experimental sessions. The experiments were conducted in agreement with Legislative Decree 27/92 and approved by the local ethics committee (Approval number 198/2013-B).

#### 2.3.2. Zymosan-Induced Edema Formation Assay

This test was performed as previously described [14]. The extract, dissolved in DMSO:saline 1:3 *v*/*v*, was administered s.c. at a dose of 100 μg/20 μL, 15 min before zymosan, into the dorsal surface of the right hind paw.

#### 2.3.3. Zymosan-Induced Thermal Hyperalgesia Assay

This assay was conducted following the previously described procedure [14]. The extract was dissolved in DMSO:saline in a ratio of 1:3 *v*/*v* and injected s.c. (100 µg/20 mL) into the paw 15 min before the zymosan. The data have been reported as maximum possible effect (MPE) determined according to the formula:

(MPE) = (post-drug latency − baseline latency)/(cut-off time − baseline latency) × 100.

#### 2.3.4. Formalin Test

The assay was performed as previously described [14]. The extract was dissolved in DMSO:saline 1:3 *v*/*v* and injected s.c. into the hind paw (100 µg/20 mL) 15 min before the formalin administration.

### 2.4. In Silico Study

The raw crystal structures of the proteins were downloaded from the RCSB protein database: AChE (6O52) [15], BChE (6EQP) [16], and Glucocorticoid receptor (4UDD) [17]. The proteins were prepared by the PrepWizard tool embedded in Maestro Schrӧdinger 2021 (Schrödinger Release 2021-2: Maestro, Schrödinger, LLC, New York, NY, USA, 2021), following a well-established protocol [18,19]. The protonation state was estimated at pH 7.0 by PropKa, and the pre-processed proteins were next minimized using OPLS4 force field.

Molecular docking experiments were performed on some of the most abundant compounds found in *J. regia* fresh fruit extract and abscisic acid. The 3D structures of quercetin, abscisic acid, citric acid, malic acid, gallic acid, 3-*p*-coumaroylquinic acid, 4-*p*-coumaroylquinic acid, ellagic acid, neochlorogenic acid, chlorogenic acid, taxifolin, ascorbic acid, catechin, and epicatechin were extracted from PubChem database and prepared by performing a geometry optimization using LigPrep module placed in Maestro (Schrödinger Release 2021-2: LigPrep, Schrödinger, LLC, New York, NY, USA, 2021), Epik at pH 7.4 (Schrödinger Release 2021-2: Epik, Schrödinger, LLC, New York, NY, USA, 2021), and OPLS4 force field. The docking grid was generated using Glide in Maestro (Schrödinger Release 2021-2: Glide, Schrödinger, LLC, New York, NY, USA, 2021), centered on the crystallographic ligand, and then used to perform molecular docking experiments. The selected compounds were docked to the prepared enzymes AChE and BChE, and abscisic acid was further docked to the glucocorticoid receptor, since it was found to interact with it [20]. The docking experiments were carried out by using Glide with the Standard Precision (SP) scoring function: the protein was kept rigid, while the ligand was set to be flexible. The best SP pose was re-docked using the eXtra Precision scoring function (XP), as described in the previously reported protocol [21]. The best docking XP poses obtained were submitted to MM-GBSA dG binding energy estimation of Prime module set in Maestro [22] (Schrödinger Release 2021-2: Prime, Schrödinger, LLC, New York, NY, USA, 2021).

### 2.5. HPLD-DAD Analysis

The identification and quantification of abscisic acid and quercetin were conducted by using an RP-HPLC, Agilent Technologies, 1100 Infinity (Waldbronn, Germany), equipped with a degasser, binary pump, automatic injector, and diode array detector (DAD). The column was a ZORBAX Eclipse Plus, C18 3.5 μm, 4.6 × 100 mm, USUXR42236, Santa Clara, CA, USA. The mobile phase was composed of (A) H_2_O 0.1% HCOOH and (B) ACN 0.1% HCOOH. The gradient used for abscisic acid was 0–3 min 95% A, 3–20 min 25% A, 20–21 min 25% A, 21–22 min 95% A. A slight change in the gradient was applied for quercetin: 0–3 min 95% A, 3–19 min 25% A, 19–20 min 25% A, 20–22 min 95% A. The flow was set to 1 mL/min, the column temperature to 30 °C, and the injection to 2 µL. Abscisic acid was detected at a wavelength of 265 nm, while quercetin was detected at 350 nm. The mobile phase was selected based on the available broad literature regarding the HPLC detection of quercetin and abscisic acid, while the gradient parameters, column temperature, flow rate, and injection volume were chosen after performing different tests to achieve a good separation of the chromatographic peaks, considering also the specifications of the used column. The wavelengths were selected by observing the highest UV absorption in a range of 250–350 nm.

The calibration curve of abscisic acid was prepared using concentrations between 0.1–100 mg L^−1^, while a range of 1–100 mg L^−1^ was used for quercetin. Both the calibration curves had an R^2^ > 0.999. The results are expressed as the median of three parallel experiments ± standard deviation (SD).

### 2.6. Statistical Analysis

The statistical analysis related to the in vivo experiments was performed by using unpaired *t*-test with Welch’s correction. GraphPad prism software (version 10.5.0, GraphPad Software Inc., San Diego, CA, USA) was used for all the analyses. Statistical significance was set at *p* < 0.05.

## 3. Results

### 3.1. Zymosan-Induced Edema Formation Assay

**Figure 1:**
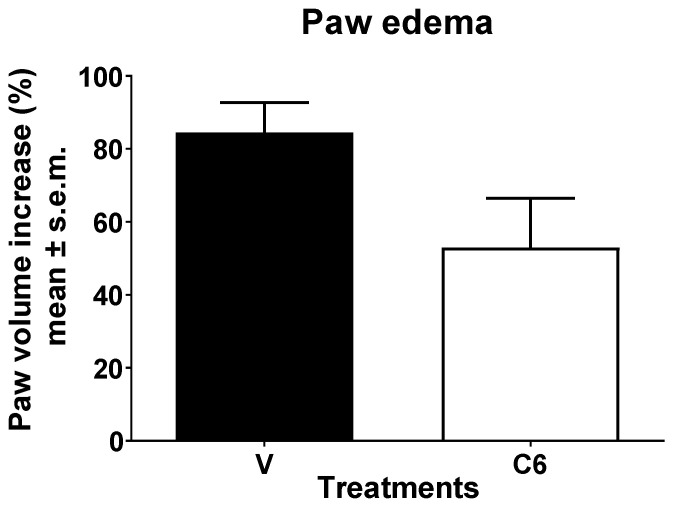
Effects induced by *J. regia* extract (C6) administered s.c. into the hind paw. Statistical analysis was performed by using unpaired *t*-test with Welch’s correction. V is for vehicle-treated animals. N = 6.

In the zymosan-induced edema formation test, mice were subjected to a pre-treatment with *J. regia* fresh fruit extract (100 µg) injected into the hind paw 15 min before the zymosan (injected in the same way). The formation of the edema was evaluated before and 4 h after the stimulus. As shown in Figure 1, a light but not significant edema reduction was induced by the extract.

### 3.2. Zymosan-Induced Thermal Hyperalgesia Assay

**Figure 2:**
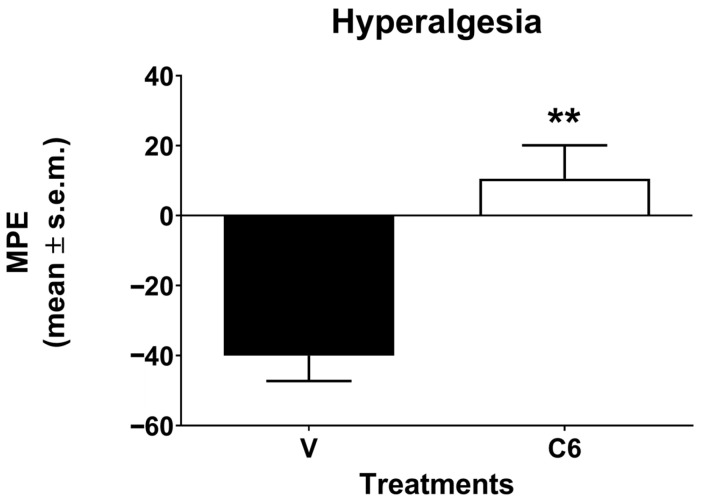
Effect induced by *J. regia* fresh fruit extract on zymosan-induced thermal hyperalgesia. Data are expressed as the maximum possible effect (MPE) according to the formula mentioned in section. Statistical analysis was performed by using unpaired *t*-test with Welch’s correction. ** is for *p* < 0.01 vs. V (vehicle-treated animals). N = 6.

The result of zymosan-induced thermal hyperalgesia was reported in Figure 2. After the injection of zymosan, a hyperalgesic effect was recorded as a decrease in nociceptive threshold to the thermal stimulus. The *J. regia* fresh fruit extract led to the reversion of the hyperalgesia induced by zymosan.

### 3.3. Formalin Test

**Figure 3:**
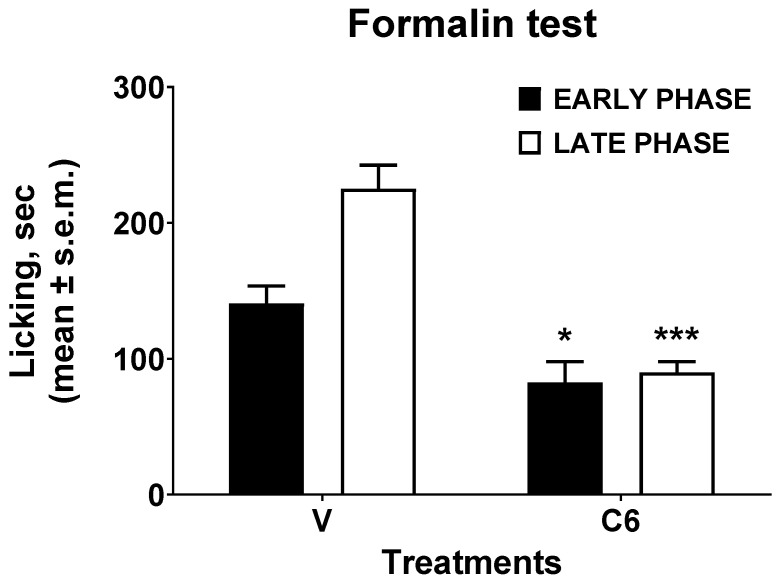
Effect induced by *J. regia* extract administered s.c. into the hind paw in formalin test. Statistical analysis was conducted by unpaired *t*-test with Welch’s correction. * is for *p* < 0.05 and *** is for *p* < 0.001 vs. V (vehicle-treated animals). N = 6.

The last in vivo assay performed to determine the analgesic effect of *J. regia* extract was the formalin test. The result was shown in Figure 3. In the early phase of the formalin test, the administration of the extract reduced the mice’s nociceptive behavioral response induced by the injected formalin. In the same way, in the late phase of the formalin test, the extract determined a more significant reduction in the licking time caused by the injected aldehyde.

### 3.4. In Silico Experiments

Since recent literature described an antinociceptive effect via non-opioid receptors and anti-inflammatory effect in acute and chronic inflammation models of aqueous and ethanolic extracts of *J. regia* leaves [5], and considering the phytochemical profile and AChE/BChE inhibition of the *J. regia* fresh fruit extract previously reported [8], we decided to explore the possible interactions of quercetin, citric acid, malic acid, gallic acid, 3-*p*-coumaroylquinic acid, 4-*p*-coumaroylquinic acid, ellagic acid, neochlorogenic acid, chlorogenic acid, taxifolin, ascorbic acid, catechin, and epicatechin on the binding pocket of two enzymes, AChE and BChE, potentially related to inflammation. Abscisic acid was also tested on the binding pocket of AChE, BChE, and the glucocorticoid receptor. Table 1 shows the best-ranked docking poses, and Table 2 shows the MM-GBSA dG binding values calculated for the best protein-ligand complexes in terms of docking scores.

Citric acid and malic acid were completely inactive in AChE and BChE in molecular docking experiments, exhibiting poor docking scores. Gallic acid was found to be slightly active in AChE and BChE in molecular docking experiments. 3-*p*-coumaroylquinic acid was found to be slightly active in AChE and quite active on BChE, but less than quercetin, ellagic acid, and neochlorogenic acid, which gave the best results in molecular docking experiments with BChE. Good results from molecular docking calculations were also obtained for catechin and taxifolin on AChE (see Table 1). However, these compounds resulted in low amounts in *J. regia* extract according to the HPLC-Q-TOF-MS analysis, which may indicate that their role in the anti-inflammatory activity of this extract is limited [8]. On the other hand, compounds such as neochlorogenic acid and ellagic acid resulted in higher amounts and showed optimal docking scores, suggesting that these active compounds might be among the key components that induce the in vivo anti-inflammatory effect of the *J. regia* extract. For compounds that were detected in major quantities in *J. regia* extract and gave favorable docking scores, the interactions with enzymatic pockets were further analyzed.

**Figure 4:**
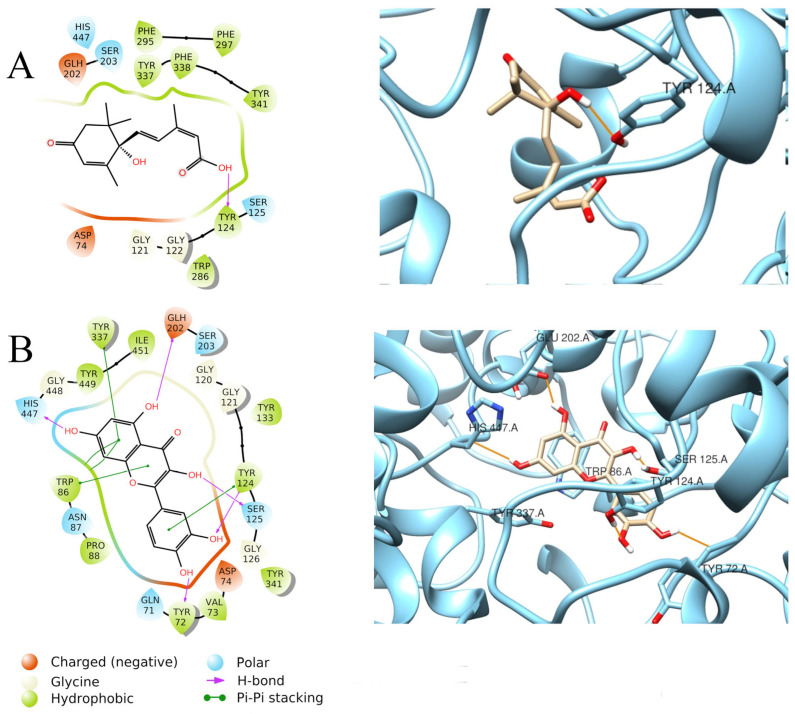
Graphical 2D representation of interactions (**left**) and binding pose (**right**) of ABA-AChE (**A**); quercetin-AChE (**B**).

**Figure 5:**
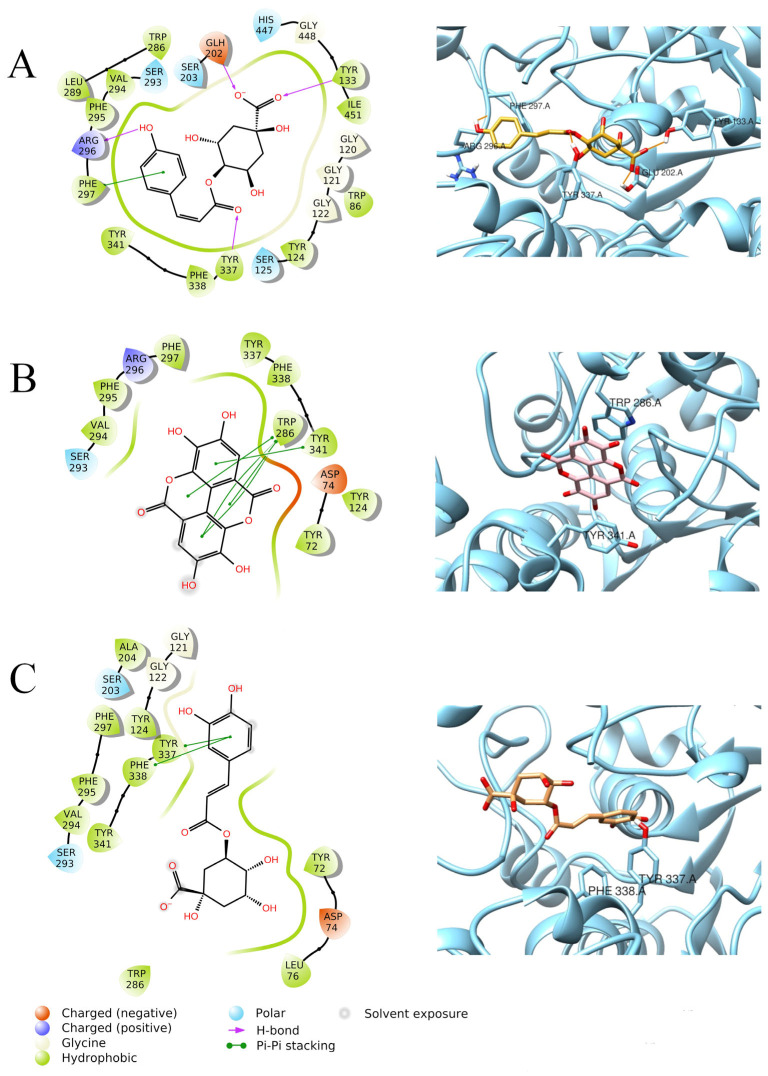
Graphical 2D representation of interactions (**left**) and binding pose (**right**) of 4-*p*-coumaroylquinic acid-AChE (**A**); ellagic acid-AChE (**B**); chlorogenic acid-AChE (**C**).

Quercetin had the best fit into the enzymatic pocket of AChE, with a docking score of −11.306 Kcal/mol. It establishes several interactions within the pocket, mainly through H-bonds with Tyr72, Ser125, Tyr124, Glu202, and His447, but it also forms π–π stacking interactions with Trp86, Tyr124, and Tyr337 (Figure 4B). This thick net of interactions may explain the better docking score of quercetin, compared to abscisic acid (−7.521 Kcal/mol), which only forms a H-bond with Tyr124 (Figure 4A). Ellagic acid also displays an optimal docking score (−9.983 Kcal/mol), due to several π–π interactions between Trp286, Tyr341 residues of AChE, and the aromatic rings in the ligand structure (Figure 5B). The optimal fitting of ellagic acid into the AChE pocket is further confirmed by the MM-GBSA dG binding value of −69.74 Kcal/mol. Similar docking scores were also found for chlorogenic acid (−9.303 Kcal/mol) and 4-*p*-coumaroylquinic acid (−9.144 Kcal/mol) when docked to AChE. Chlorogenic acid establishes π–π interactions with Tyr337 and Phe338 (Figure 5C), while 4-*p*-coumaroylquinic acid interacts with Tyr133, Tyr337, Arg296, and Glu202 through H-bonds and establishes a π–π stacking with Phe297 (Figure 5A).

**Figure 6:**
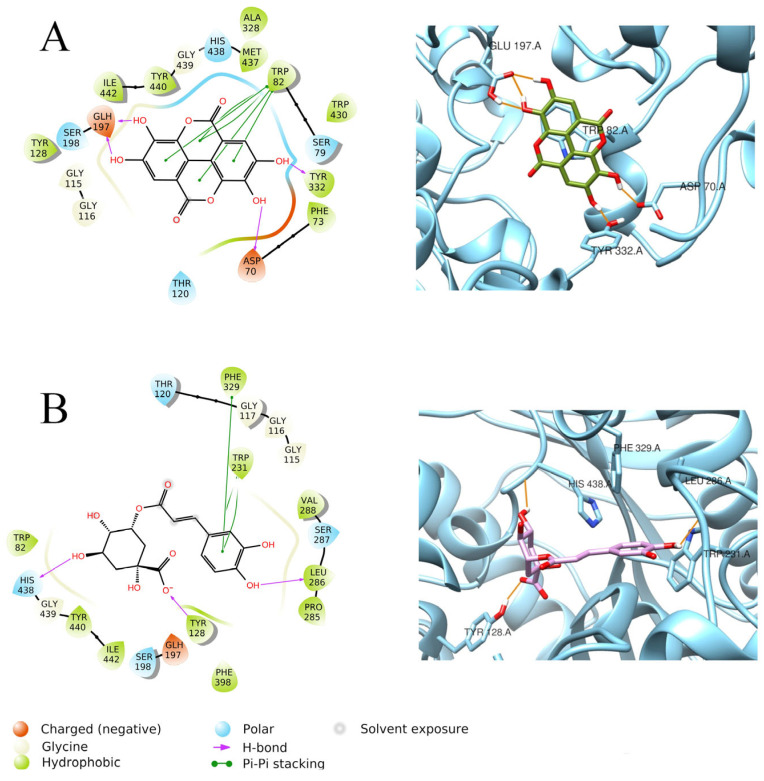
Graphical 2D representation of interactions (**left**) and binding pose (**right**) of ellagic acid-BChE (**A**); neochlorogenic acid-BChE (**B**).

Neochlorogenic acid and ellagic acid gave the best results on BChE. Neochlorogenic acid, with a docking score of −10.292 Kcal/mol, displayed three H-bonds with His438, Tyr128, and Leu286 and π–π interactions with Phe329 and Trp231 (Figure 6B), even though it resulted in a weak MM-GBSA dG binding value (−10.77 Kcal/mol). Ellagic acid showed a similar docking score of −10.054 Kcal/mol, but a more favorable MM-GBSA dG binding value (−56.18 Kcal/mol), ascribable to H-bonds established with Tyr332, Asp70, Gln197, and a π–π stacking with Trp82 inside the enzymatic pocket of BChE (Figure 6A).

**Figure 7:**
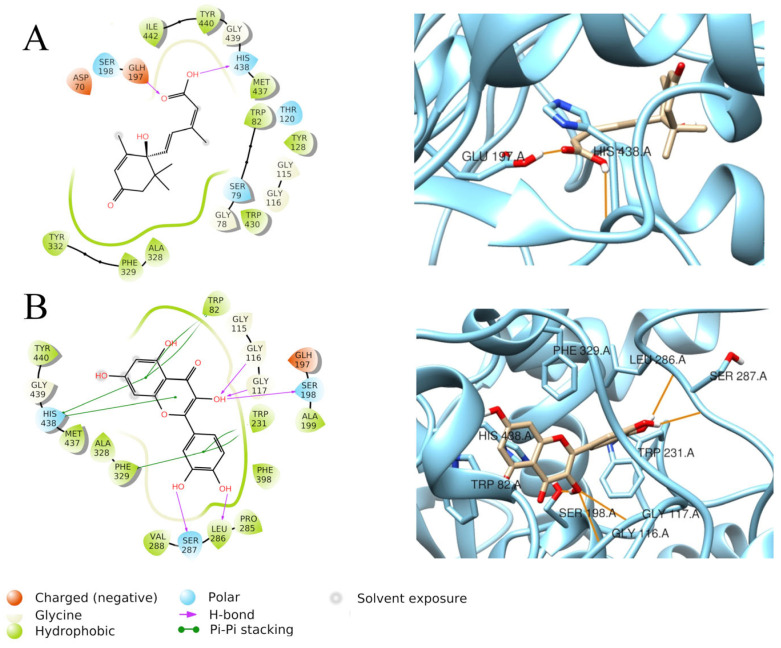
Graphical 2D representation of interactions (**left**) and binding pose (**right**) of ABA-BChE (**A**); quercetin-BChE (**B**).

Quercetin also showed a fine docking score when docked to BChE (−9.894 Kcal/mol), and it established H-bonds with Gly116, Gly117, Ser198, Leu286, and Ser287 but also a net of π–π stacking interactions with Trp82, Trp231, Phe329, and His438 due to the presence of aromatic rings inside the quercetin structure (Figure 7B). Quercetin also gave a notable MM-GBSA dG binding value of −47.06 Kcal/mol. On the other side, abscisic acid showed, again, only two H-bonds with Glu197 and His438, which supports its lower docking score when docked to BChE (−6.640 Kcal/mol) (Figure 7A).

**Figure 8:**
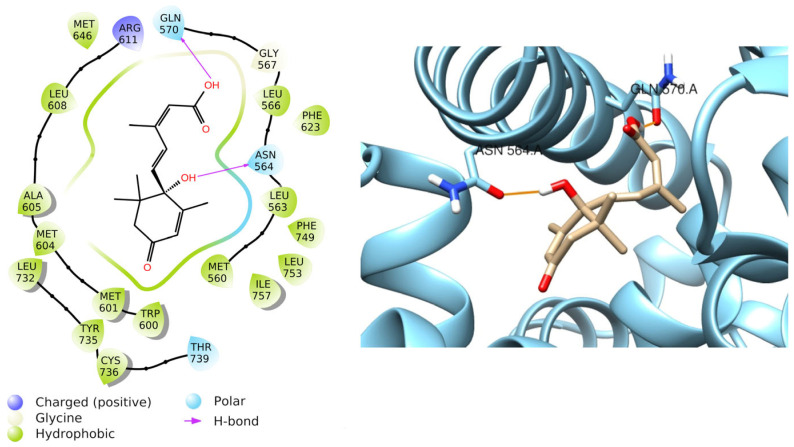
Graphical 2D representation of interactions (**left**) and binding pose (**right**) of ABA-Glucocorticoid receptor.

Relying on the results obtained from previous literature [20], abscisic acid was also docked to the glucocorticoid receptor and resulted in a favorable docking score equal to −8.772 Kcal/mol. Furthermore, it forms two H-bonds with Asn564 and Gln570 (Figure 8), with a MM-GBSA dG binding equal to −50.41 Kcal/mol, confirming the stability of the protein-ligand complex.

Therefore, this result indicates that abscisic acid may be involved in the downstream signaling of the glucocorticoid receptor during an inflammatory state.

### 3.5. Quantification of Quercetin and Abscisic Acid

**Figure 9:**
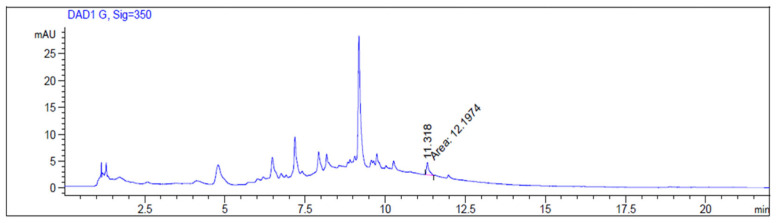
HPLC-DAD chromatogram of *J. regia* fresh fruit extract at 350 nm. The indicated peak corresponds to quercetin.

A *J. regia* fresh fruit extract was prepared, and two compounds, specifically quercetin and abscisic acid, were quantified. Quercetin is a flavonoid characterized by antioxidant, antidiabetic, antimicrobial, and anticancer activities [23]. The quantification of quercetin was performed by HPLC-DAD using its analytical standard (external standard), observing a total amount of 0.246 ± 0.003 mg/g DE (dried extract) (Figure 9).

**Figure 10:**
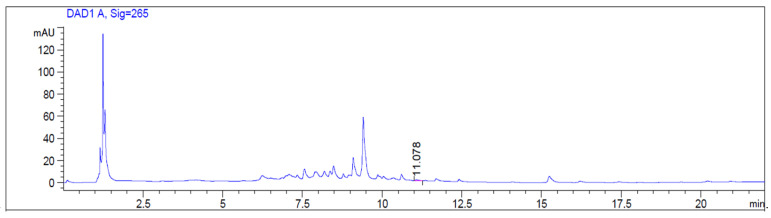
HPLC-DAD chromatogram of *J. regia* fresh fruit extract at 265 nm. The indicated peak corresponds to abscisic acid.

Abscisic acid (ABA) belongs to terpenoids and plays an important role in adaptation to abiotic environmental stress, development of seeds, and germination [24,25]. The metabolism of some hormones, like abscisic acid, is subjected to changes in concentrations during fruit ripening, with a decrease in levels; it can be considered a ripening control factor [26,27,28]. For the preparation of *J. regia* extract, immature walnut fruits were used. Thus, the identification and quantification of this compound, not investigated in our previous work, was conducted. Indeed, dietary ABA was effective in the reduction of gut inflammation by regulating T cell distribution and the expression of adhesion molecules [29]. A quantity of 0.036 ± 0.004 mg/g DE was detected in *J. regia* fresh fruit extract (Figure 10).

## 4. Discussion

In this work, the potential anti-inflammatory and analgesic effects of a *J. regia* fresh fruit extract were determined by in vivo assays. As previously reported, the extract contained relevant quantities of gallic acid and derivatives, coumaric acid derivatives, caffeoylquinic acids, and ellagic acid. Quercetin and different quercetin glycosides were also present. Several studies reported the analgesic and anti-inflammatory properties of these kinds of compounds, justifying our interest in the assessment of the potential anti-inflammatory effect of the whole extract. For instance, Krogh et al. developed a QSAR model to predict the analgesic potency of gallic acid analogues [30]. Yang et al. evaluated the analgesic effect of gallic acid on neuropathic pain in rats with chronic constriction injury. Here, it was able to inhibit the activation of the satellite glial cells in the dorsal root ganglia, alleviating thermal and mechanical hyperalgesia by downregulating P2X7 receptor expression, reducing the mature TACE release, inhibiting the expression of TNF-α, and suppressing the NF-κB/STAT3 signaling pathway [31]. In another study, gallic acid had ameliorative effects in rats with DIC-induced renal injury thanks to the modulation of oxidative stress and by inhibiting the inflammatory response [32]. The anti-inflammatory effect of *p*-coumaric acid was assessed by Pragasam et al. in rats with MSU crystal-induced inflammation [33]. Moreover, Guilherme et al. intercalated coumaric acid into LDH (layered double hydroxide), which demonstrated impressive results in the tail-flick test, increasing the duration of analgesia by about 1.7–1.8 times in comparison with coumaric acid or indomethacin [34]. Quercetin nanoparticle gel was tested on rats in which osteoarthritis was induced. A decrease in the inflammation process was obtained by reducing the edema volume [35]. Another study on the anti-inflammatory effect of quercetin revealed a reduction in NO, IL-6, and NF-kB in lipopolysaccharide (LPS)-stimulated RAW264.7 macrophages. Furthermore, the oral administration of quercetin, galangin, or a combination of the two compounds was tested on a mouse model with atopic dermatitis induced by 2,4-dinitrochlorobenzene. The two flavanols caused a decrease in inflammation, while this effect was amplified using quercetin in combination with galangin [36]. Chlorogenic acid was also studied for its inflammatory properties; it blocks the production and secretion of inflammatory mediators (e.g., NO, TNF-α, PGE2, and COX-2) essential for the beginning and progression of inflammation [37]. Furthermore, chlorogenic acid showed anti-edematogenic and antinociceptive effects, respectively, on animal models of carrageenin-induced inflammation and formalin-induced pain [38]. According to Kim et al., neochlorogenic acid displayed a decrease in LPS-induced NO production through the suppression of iNOS and COX-2 protein expression and the production of pro-inflammatory cytokines (e.g., TNF-α and IL-1β) in BV2 microglia cells. Moreover, neochlorogenic acid inhibited phosphorylated p38 MAPK and NF-jB p65 in activated microglia [39].

In this work, three different assays were performed on animal models. In the zymosan-induced edema formation test, a light but not significant edema reduction was determined by the administration of the extract. In the zymosan-induced thermal hyperalgesia assay, the *J. regia* fresh fruit extract induced the reversion of the hyperalgesia caused by zymosan. The last in vivo assay was the formalin test. In the early phase, a reduction of the mice’s nociceptive behavioral response induced by the injected formalin was observed after the administration of the extract. In the late phase of the test, the extract determined a higher reduction in the licking time caused by the aldehyde. Overall, these data indicate a potent anti-inflammatory effect in vivo of the *J. regia* fresh fruit extract after s.c. administration, which suggests a strong stability in human plasma and a potent analgesic activity in the early and late phases of the formalin test.

Since the *J. regia* fresh fruit extract exhibited an inhibition effect on AChE and BChE in vitro, these enzymes have been considered in the in silico study. Indeed, their activities regulate the “cholinergic tone”, the total capacity for ACh hydrolysis in the human body; thus, their inhibitors can modify the cholinergic tone. ACh can stop inflammation through NF*k*b signaling system; consequently, the AChE inhibitors might heighten the ACh levels, limit the inflammation in human tissues, and send messages to the central nervous system through the vagus nerve informing the brain about the modification of the cholinergic tone and inflammation levels [40,41,42].

The in silico determination was conducted by evaluating the potential interactions of the main components of the extract, in particular organic acids, phenolic acids, and flavonoids, on the binding pocket of AChE and BChE. Furthermore, along with these compounds, abscisic acid was also considered in this study. Indeed, dietary ABA resulted in being effective in the reduction of gut inflammation [29]. Guri et al. observed that the supplementation with dietary ABA ameliorated the disease and colonic inflammatory lesions in experimental inflammatory bowel disease through a T cell PPARγ-dependent mechanism [43]. Its presence in the extract was not previously evaluated, but considering its role in adaptation, development of seeds, and germination [24,25], this compound could be part of the extract. Citric acid, malic acid, gallic acid, 3-*p*-coumaroylquinic acid, 4-*p*-coumaroylquinic acid, ellagic acid, neochlorogenic acid, chlorogenic acid, quercetin, and abscisic acid were docked to AChE and BChE. The docking score of abscisic acid was also determined when docked with the glucocorticoid receptor. Quercetin showed the best docking score and a thick net of interactions with the AChE catalytic pocket, along with a favourable dG MM-GBSA value (−27.29 Kcal/mol). Also, chlorogenic acid (1.01 mg/g DE (dried extract)), ellagic acid (1.4 mg/g DE), and 4-*p*-coumaroylquinic acid (1.5 mg/g DE) showed high docking scores with the AChE catalytic pocket. Neochlorogenic acid (1.9 mg/g DE) and ellagic acid gave the highest docking score with BChE, followed by quercetin. The quantification data are reported and described in our previous work [8]. The results from molecular docking are in accordance with the in silico study conducted by Nazir et al., who pointed out higher cholinesterase inhibitory activities for ellagic acid and chlorogenic acid, whereas a weaker inhibitory activity was found for gallic acid [44]. Furthermore, Liao et al. investigated the inhibition of quercetin on AChE through computational methods. They highlighted a relevant inhibitory activity of quercetin, which interacts with the enzymatic pocket of AChE, with Tyr337, Gln202, Asp74, and Tyr124 residues, agreeing with our in silico results [45]. Abscisic acid was found slightly active on AChE and BChE, but when docked to the glucocorticoid receptor showed a favorable score and a good stability of the protein-ligand complex. Considering these results, it may be involved in the downstream signaling of the glucocorticoid receptor during an inflammatory state.

The presence of abscisic acid and quercetin in the extract was assessed by HPLC-DAD analysis and further quantified, obtaining 0.036 ± 0.004 mg/g DE and 0.246 ± 0.003 mg/g DE for both of them, respectively.

## 5. Conclusions

To summarize, acute and chronic inflammatory states are common features of diverse human diseases such as dysmetabolic and neurodegenerative disorders. Some key enzymes are deeply involved in the regulation and control of their development and should be taken into consideration when thinking of a target therapy, both pharmacological and palliative. Overall, the data obtained in this study showed high anti-inflammatory activity in vivo of the *J. regia* fresh fruit extract in CD-1 mice. Some of the compounds found in the extract were tested in silico to determine their potential binding to AChE and BChE, showing that quercetin and neochlorogenic acid had the highest docking scores when docked with AChE and BChE, respectively, while abscisic acid has a good binding profile on the glucocorticoid receptor.

Thus, this research highlights the possibility of developing a nutraceutical with strong anti-inflammatory activity thanks to the presence of relevant compounds able to bind pivotal enzymes involved in serious human diseases. The development of a suitable technological formulation for this extract will be the next step of our ongoing study.

## Figures and Tables

**Table 1:** Best-ranked docking poses obtained for each protein-ligand system (Kcal/mol).

Cps	AChE SP	AChE XP	BChE SP	BChE XP	Glucocorticoid Receptor SP	Glucocorticoid Receptor XP
Abscisic acid	−6.253	−7.521	−6.620	−6.640	−7.428	−8.772
Quercetin	−7.652	−11.306	−8.336	−9.894	-	-
Citric acid	−4.474	+0.012	−5.393	−3.297	-	-
Malic acid	−4.496	−3.411	−4.032	−3.742	-	-
Gallic acid	−6.299	−7.368	−6.273	−5.923	-	-
3-*p*-coumaroylquinic acid	−7.424	−6.275	−7.625	−9.440	-	-
4-*p*-coumaroylquinic acid	−7.353	−9.144	−6.796	−7.312	-	-
Ellagic acid	−8.451	−9.983	−8.058	−10.054	-	-
Neochlorogenic acid	−7.718	−6.624	−7.811	−10.292	-	-
Chlorogenic acid	−7.832	−9.303	−5.883	−8.917	-	-
Taxifolin	−7.873	−10.760	−7.381	−9.603	-	-
Ascorbic acid	−4.619	−6.237	−4.889	−4.154	-	-
Catechin	−7.820	−11.077	−8.146	−8.788	-	-
Epicatechin	−8.491	−8.031	−8.706	−9.325	-	-

**Table 2:** ΔG MM-GBSA values obtained for each protein-ligand system (Kcal/mol).

Cps	AChE	BChE	Glucocorticoid Receptor
Abscisic acid	−14.90	−22.90	−50.41
Quercetin	−27.29	−47.06	-
4-*p*-coumaroylquinic acid	−22.20	-	-
Ellagic acid	−69.74	−56.18	-
Neochlorogenic acid	-	−10.77	-
Chlorogenic acid	−32.57	-	-
Taxifolin	−43.41	−33.76	-
Ascorbic acid	−6.15	−5.61	-
Catechin	−49.31	−31.80	-
Epicatechin	−44.87	−35.75	-

## Data Availability

The original contributions presented in this study are included in the article. Further inquiries can be directed to the corresponding author.

## Abbreviations

The following abbreviations are used in this manuscript:

BPH	Benign Prostatic Hyperplasia
TNF-α	Tumor Necrosis Factor alpha
IL	InterLeukin
NF-κB	Nuclear Factor kappa-light-chain-enhancer of activated B cells
COX-2	Cyclooxygenase-2
CXC-R1	C-X-C Chemokine Receptor type 1
RP-HPLC	Reverse Phase High-Performance Liquid Chromatography
DAD	Diode Array Detector
SD	Standard Deviation
OPLS	Optimized Potentials for Liquid Simulations
ACh	AcetylCholine
AChE	AcetylCholinEsterase
BChE	ButyrylCholinEsterase
SP	Standard Precision
XP	eXtra Precision
s.c.	SubCutaneous
DMSO	DiMethyl SulfOxide
PWL	Paw Withdrawal Latency
MPE	Maximum Possible Effect
DE	Dried Extract
ABA	Abscisic Acid
PPARγ	Peroxisome Proliferator-Activated Receptor gamma
CPS	Compounds
QSAR	Quantitative Structure-Activity Relationship
Tyr	Tyrosine
Ser	Serine
Glu	Glutamic acid
His	Histidine
Trp	Tryptophan
Gly	Glycine
Leu	Leucine
Phe	Phenylalanine
Asn	Asparagine
Gln	Glutamine
Arg	Arginine
Asp	Aspartic acid
TACE	TNF-α converting enzyme
STAT3	Signal Transducer and Activator of Transcription 3
DIC	Diclofenac
MSU	Monosodium Urate
LDH	Layered Double Hydroxide
NO	Nitric Oxide
LPS	Lipopolysaccharide
PGE2	Prostaglandin E2
iNOS	Inducible Nitric Oxide Synthase
MAPK	Mitogen-Activated Protein Kinase
